# Intradural Extramedullary Concurrent Schwannoma and Meningothelial Hyperplasia at C2-C3 Cervical Vertebrae: A Case Report and Review of Literature

**DOI:** 10.1155/2022/1087918

**Published:** 2022-05-05

**Authors:** Rayan Rammal, Daniel F. Marker, Rana Naous

**Affiliations:** ^1^Department of Pathology, University of Pittsburgh Medical Center, Pittsburgh, PA, USA; ^2^Department of Pathology, University of Pittsburgh Medical Center, Division of Neuropathology, UPMC Presbyterian, Pittsburgh, PA, USA; ^3^Department of Pathology, University of Pittsburgh Medical Center, Division of Bone and Soft Tissue Pathology, UPMC Shadyside, Pittsburgh, PA, USA

## Abstract

Concomitant schwannomas and benign meningothelial proliferations, including meningothelial hyperplasia or meningioma, rarely occur at the same location outside the setting of neurofibromatosis. Herein, we present a rare case of concurrent schwannoma and benign meningothelial hyperplasia concomitantly occurring in the cervical spine of a 69-year-old male patient with no history of any genetic disorder.

## 1. Introduction

Schwannomas and meningiomas are the two most common spinal tumors, representing 30% and 25% of spinal cord tumors, respectively [1]. In the spinal cord, the majority of schwannomas (75%) arise in the intradural extramedullary portion with the high cervical region being the most frequent site of involvement (15% arise exclusively in the extradural area and the rest have both intradural and extradural components), while meningiomas are found in the thoracic spine and less commonly in the cervical spine (15%) [1]. Multiple concomitant spinal cord tumors are uncommon (<10%) and are usually associated with neurofibromatosis type 2 (NF2) with about 21% of schwannomas in NF2 patients being associated with concurrent meningiomas [2, 3] or von Hippel Lindau disease [1, 3, 4]. Concomitant meningiomas with variant histopathologic types, as opposed to the conventional type, are even rarer (0.3%) [1, 4]. Only seven cases with simultaneous occurrence of schwannoma and meningioma at the same cervical spine level and without associated genetic disorder have been reported in the literature [1–6].

Meningothelial hyperplasia is a poorly characterized entity that is thought to be reactive in nature and is often seen in association with advanced age, chronic kidney disease, trauma, hemorrhage, and neoplasia [7, 8]. In the latter setting, meningothelial hyperplasia has been reported to be particularly prominent adjacent to optic nerve pilocytic astrocytoma, where it can be misdiagnosed as orbital meningioma [8]. Meningothelial/arachnoid proliferation appears to also be quite exuberant in bilateral acoustic neurofibromas and has additionally been reported to be florid in the cerebellopontine angle/petrous temporal bone around schwannomas thus resulting in the formation of multiple “micromeningiomas” [9]. Meningothelial hyperplasia may cause diagnostic challenges as it has overlapping histologic features with meningioma [8].

Herein, we present the case of concomitant schwannoma and a benign meningothelial hyperplasia arising in the cervical spine of a 69-year-old man.

## 2. Case Presentation

A 69-year-old male patient presents with a chief complaint of neck pain of several weeks, radiating to the head and right shoulder. The pain is reported to be intermittent, burning in quality, associated with weakness and numbness especially in the right hand. The patient has a past medical history of hypertension, hyperlipidemia, benign prostatic hyperplasia, DVT/pulmonary embolus in the past year for which he is on anticoagulant therapy, and a surgical history of bilateral knee replacement within the past two years. The patient has no history of neurofibromatosis or stigmata of another genetic disorder. Upon physical examination, the patient has right hemiparesis (-4/5) that has mildly progressed compared to prior examination. MRI of the cervical spine (performed at an outside institution) reveals a T1 hypointense and T2 heterogeneously hyperintense extramedullary mass causing impingement upon the right side of the spinal cord which is flattened and displayed to the left at the C2-C3 level. The mass measures approximately 35 mm in transverse diameter by 13 mm in AP diameter by 18 mm in craniocaudal diameter and laterally extends through the right C2-C3 neural foramen. The radiologic impression is that of a nerve sheath tumor of either benign or malignant nature (no images available). Surgery is recommended, and the patient undergoes a C2-C3 laminectomy at an outside institution with resection of the tumor. Intraoperatively, the tumor is seen on the right side of the canal with the cord. Dorsolaterally, the tumor appears to blend in with the dura at its base. The junction is coagulated with bipolar cautery. Then, some of the tumor is debulked along the base, allowing it to shrink away from the cord laterally. Working around the superior pole of the tumor then reveals rootlets that appear to be joining the tumor to the right superior pole along its posterior aspect. Attempting to dissect to release it reveals that the rootlets were not just adherent to the capsule but rather growing into the tumor. The rootlets are hence cauterized and amputated at base. The tumor is removed in piecemeal fashion, and the tissue is sent to pathology. Grossly, the specimen consists of multiple pieces of light tan, firm tissue measuring in aggregate (2.0 × 1.6 × 0.3 cm).

**Figure 1:**
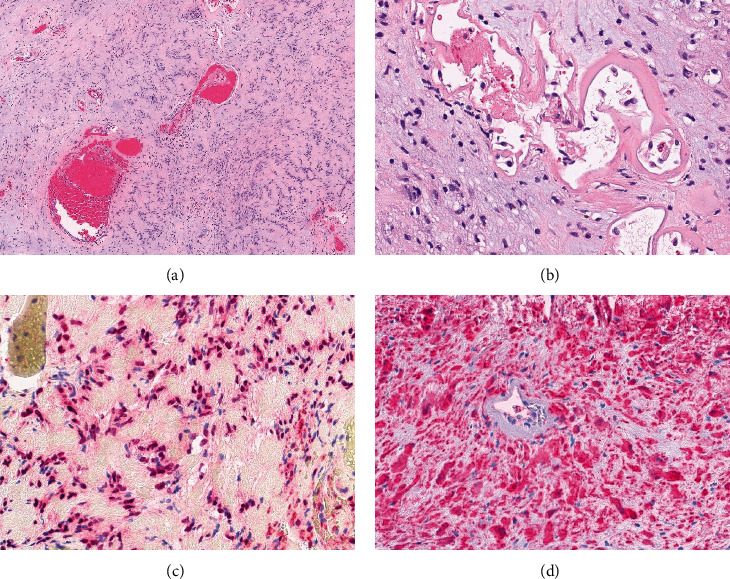
(a) Low-power examination reveals Schwannian cells arranged in loose fascicles, surrounded by eosinophilic stroma with Verocay bodies (40x). (b) Higher power examination shows the bland Schwannian cells set in a loose collagenous stroma with hyalinized vessels (200x). Schwannian cells are positive for (c) SOX-10 (200x) and (d) S100 (200x).

**Figure 2:**
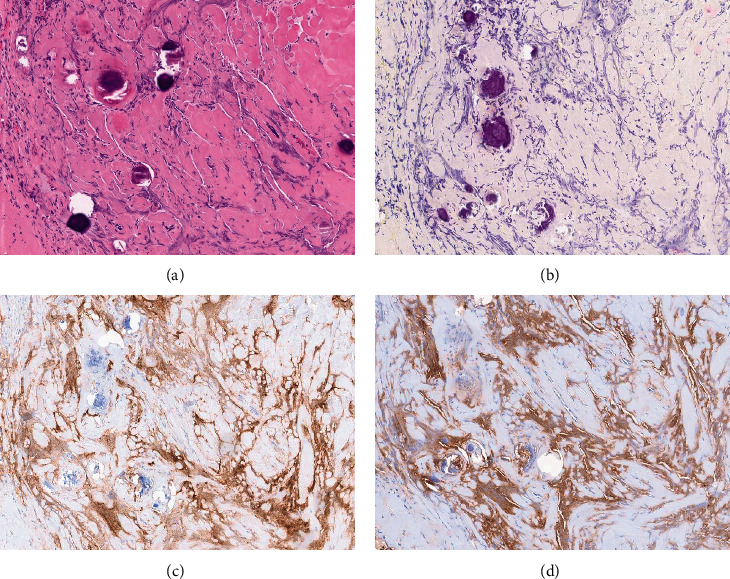
(a) Separate fibrous fragment with psammomatous calcifications and vague bland spindled to ovoid cells is shown. This fragment is negative for (b) SOX-10 and positive for (c) EMA and (d) SSTR2A (all images at 100x).

The slides are sent to our institution for review. Microscopic examination reveals fragments of a benign nerve sheath tumor comprised of Schwannian cells arranged in loose fascicles set in an eosinophilic stroma with occasional hyalinized vessels and Verocay bodies, compatible with Schwannoma (Figures 1(a) and 1(b)). Also present was a small separate fibrous fragment with psammomatous calcifications and vaguely nested bland spindled to ovoid cells with focal crush artifact (Figure 2(a)). No necrosis or mitotic figures are identified. Immunohistochemical stains are performed and show that the tumor cells are diffusely positive for S100 and SOX-10 in the Schwannoma component (Figures 1(c) and 1(d)). The small fibrous fragment, on the other hand, is negative for SOX-10 (Figure 2(b)) and shows weak staining for S100 while PR and CD34 show patchy weak staining and EMA and SSTR2A are positive (Figures 2(c) and 2(d)) thus compatible with a meningothelial origin. HMB-45, Melan A, and STAT6 are negative.

The overall histopathological and immunohistochemical findings are compatible with Schwannoma with a small focus of benign meningothelial proliferation compatible with benign meningothelial hyperplasia. MRI with and without contrast performed three months after the surgery shows postoperative changes of subtotal resection of the enhancing mass centered about the right C2-C3 foramen, with a residual 21 × 10 × 16 mm (axial long axis × axial short axis × craniocaudal) right neural foraminal mass and significant improvement in the spinal cord compression. At six month postsurgery, MRI demonstrates improved postsurgical changes with redemonstration of the residual spinal enhancing mass at the right C2-C3 neural foramen extending into the spinal canal with minimal mass effect on the spinal cord on the right aspect of the cord at this level. No further available clinical data could be obtained due to the patient being lost to follow-up.

## 3. Discussion

While previous cases of simultaneous schwannoma and meningiomas arising at the same level of cervical spine have been reported, concomitant schwannoma and meningothelial hyperplasia have not been previously reported at the same location in any site. In the present case, a 69-year-old man presents with an intradural extramedullary mass at C2-C3. Pathology shows a schwannoma with a separate fragment of meningothelial proliferation, most compatible with meningothelial hyperplasia. The previously reported seven cases of concurrent schwannoma and meningioma occurring at the same spinal level consisted of a predominantly extradural component of the mass which was a schwannoma in addition to a smaller intradural extramedullary component which represented a meningioma [1–3, 5, 6], except for one case where both tumors were intradural extramedullary [4]. The schwannoma and meningioma in each of these cases formed distinct lesions both macroscopically and microscopically [1–6]. On the other hand, most cases of meningothelial hyperplasia are detected only microscopically [6]. Meningothelial hyperplasia is believed to be a reactive process characterized by a proliferation of arachnoidal cap cells that often has a multicentric growth pattern and at least focally reaches a thickness of 10 or more cell layers [7]. It can be up to 100 cell layers thick and several millimeters in the greatest dimension but has a discontinuous growth pattern with patches of hyperplastic nests and never invades the dura [6, 8]. Immunohistochemistry cannot distinguish between meningothelial hyperplasia and meningioma as both share similar expression profiles.

In contrast to the previously reported cases, in our case, only one mass was discernible macroscopically, thus favoring meningothelial hyperplasia over a concomitant meningioma. Among the eleven reported cases of meningothelial hyperplasia, 64% overlapped immunohistochemically with meningiomas in terms of progesterone receptor (PR) expression and two cases showed chromosomal gains, in contrast to normal arachnoidal cap cells that were negative for PR and showed no chromosomal abnormalities [8]. Furthermore, meningothelial hyperplasia lacked evidence of NF2 and 4.1B gene deletions or loss of their protein products, merlin, and protein 4.1B, while NF2 and protein 4.1B losses were previously found in the majority of meningiomas as reported by Perry et al. [8], who also note that even though their data support the notion that meningothelial hyperplasia is a reactive process that is often difficult to distinguish from meningioma, it may potentially represent a preneoplastic precursor in some instances [8]. While no association between neurofibromatosis and meningothelial hyperplasia has been reported in humans, in a mouse model of optic nerve glioma resulting from neurofibromatosis 1/NF1 inactivation in astrocytes, a preneoplastic hyperproliferative state was observed in the evolution of these tumors further suggesting the possible preneoplastic nature of this proliferation. This hypothesis, however, needs to be further tested [10]. Overall, it is safe to conclude that whenever it is difficult to clinically distinguish a meningioma from meningothelial hyperplasia, lack of the aforementioned genetic alterations would favor meningothelial hyperplasia over meningioma [8]. From a surgical standpoint, this distinction has important clinical implications since a hyperplastic proliferation is regarded as a self-limited process while a neoplastic process has the capacity to grow and cause neurologic complications necessitating therapeutic intervention [8]. No additional treatment has been reportedly given for meningothelial hyperplasia, such cases being usually discovered incidentally at the microscopic level.

When it comes to cases of concomitant schwannoma and meningioma of the cervical spine without associated syndromes, based on the available literature, clinical follow-up was reported on two cases with no tumor recurrence 24 and 6 months after surgery [5, 6]. No further treatment beyond the original surgical resection was mentioned to be given. An additional case of simultaneously occurring schwannoma and meningioma at the cerebellopontine angle, without an associated syndrome, showed no evidence of recurrence 9 years after the initial surgery [11].

For cervical schwannomas arising at the high cervical levels (C1-C3), microneurosurgical management using a standard midline posterior approach, which was suggested to be the most suitable type of approach for these tumors, in combination with proper neuroradiological observation and pathological appreciations can give satisfying neurological outcomes without extensive bone removal or soft tissue manipulation [12]. Complete tumor removal can be achieved with preservation of the nerve root in most cases [12]. On long-term follow-up and in the largest series consisting of fifteen cases, all patients did not develop recurrence of their tumor [12]. While postoperative complications occurred in a minority of cases (three patients), consisting of CSF leak or wound healing problems, the remainder of the patients showed postoperative improvement in their symptoms and returned to normal function within a few months [12].

Spinal meningiomas are usually benign slow-growing tumors that respond favorably to surgical excision, especially complete resection, with low complication rates and a good long-term functional outcome [13, 14]. A longer duration of symptoms, however, has been associated with poorer neurological outcome after surgery [13]. Early surgical operation and early detection by imaging modality are hence important [13]. In cases of high grade tumors or tumors that invade the pia/arachnoid, the prognosis tends to be worse and adjuvant therapies must be considered (stereotactic radiosurgery, radiotherapy, and/or chemotherapy) [13]. While the primary treatment of spinal meningiomas remains surgery, with complete resection of the tumor achievable in most cases, preserving neurological function takes precedence over complete removal of the tumor [15]. The goal of surgery is primarily complete safe tumor removal and decompression of the spinal cord [13, 14]. A preoperative surgical grading system (Simpson grade) appeared to be helpful when considering the surgical strategy and it appears that a lower preoperative surgical grade resulted in better functional outcome whereas a higher preoperative surgical grade was significantly associated with insufficient tumor removal [14]. Although spinal meningiomas have a low recurrence rate, some reports have suggested high tumor recurrence rates in patients aged younger than 50 years at the time of surgery and in patients with long follow-up periods even after complete resection (Simpson grade II resection). The factors leading to recurrence after surgery include young age of the patient, subtotal resection of the lesion, calcification, extradural attachment, multiplicity of lesions, and anterior/ventral location of the tumor [14]. Of note, the multiplicity of lesions becomes an important factor especially in patients with associated syndromes such as NF2.

Cervical spine meningiomas constitute a very small proportion of spinal schwannomas. The largest case series consists of twenty-two cases of cervical spine meningiomas [16]. The functional outcome improvement rate after surgery in this study was very good and comparable to the improvement rates generally described for spinal schwannomas [16]. Two cases (9.1%) of attempted subtotal resection (Simpson grade IV) had many common features such as the extension of more than two levels in the craniocaudal direction, severe adhesion to the spinal cord, and presence of calcifications. Interestingly, those two patients had no recurrence of their tumor, which is significantly lower than previously reported in the literature; however, limitations of this study by Lee et al. include the small case number as well as short follow-up time [16]. One of these patients underwent adjuvant radiotherapy ten months after surgery. The overall recurrence rate in this series was 9.1% (2 patients), the patients having received Simpson grade II and Simpson grade III resection, respectively. Despite tumor recurrence, the latter patients did well without additional therapy [16]. Because of the implications on outcomes and mostly on treatment modalities, the distinction between a schwannoma and meningioma, concurrent schwannoma and meningioma, and concurrent schwannoma and meningothelial hyperplasia becomes important.

Pathogenetically, schwannomas are believed to arise from Schwann cells of the dorsal root nerves while meningiomas are thought to arise from the arachnoid membrane/cap cells [2, 6]. There are multiple theories to explain the occurrence of meningiomas and schwannomas in the same location in a patient. The first theory is that since these two tumors are common in the spinal cord, they could develop coincidentally at the same location [1, 2, 6]. The second theory is that these tumors may separately differentiate from a common mesenchymal progenitor cell at the same location [1, 6]. The third theory is that a preexisting tumor (schwannoma) alters the microenvironment, facilitating the formation of another tumor (meningioma) through expression of tumorigenesis-related cytokines, such as EGF-like molecules [1–3, 6]. This could partially explain the difference in volume/size of the two tumors whereby schwannoma tends to be larger compared to the meningioma [1].

Regarding the latter theory, reactive meningothelial hyperplasia caused by a preexisting tumor is a consideration in our case. In fact, Geddes et al. proposed it as a plausible mechanism for reactive meningothelial hyperplasia occurring in the vicinity of a schwannoma [9]. Even though it has been suggested that meningothelial hyperplasia is reactive in nature, it remains unclear whether these hyperplastic lesions are preneoplastic and if they could represent a precursor stage in meningioma tumorigenesis [8]. In our case, it is possible that the schwannoma has incited the meningothelial hyperplasia through microenvironmental factors. The purpose of this case report is to shed light on the rare cooccurrence of schwannoma and meningothelial hyperplasia and emphasize the importance of meticulous preoperative clinical and radiologic evaluation so as not to miss any possible coexisting components, whether reactive or (pre)neoplastic, within schwannomas. Additional studies are needed to explore the pathogenesis of such occurrences.

## 4. Conclusion

We report the case of a concomitant schwannoma and meningothelial hyperplasia present within the high cervical spine of a 69-year-old man with no history of any genetic disorder. This is a rare phenomenon of uncertain biologic nature for which the underlying mechanism remains to be fully elucidated.

## Data Availability

Data is available upon request from the corresponding author.
